# Small RNAs Targeting Transcription Start Site Induce Heparanase Silencing through Interference with Transcription Initiation in Human Cancer Cells

**DOI:** 10.1371/journal.pone.0031379

**Published:** 2012-02-20

**Authors:** Guosong Jiang, Liduan Zheng, Jiarui Pu, Hong Mei, Jun Zhao, Kai Huang, Fuqing Zeng, Qiangsong Tong

**Affiliations:** 1 Department of Surgery, Union Hospital of Tongji Medical College, Huazhong University of Science and Technology, Wuhan, Hubei Province, China; 2 Department of Pathology, Union Hospital of Tongji Medical College, Huazhong University of Science and Technology, Wuhan, Hubei Province, China; 3 Human Disease Related Gene Research Group, Union Hospital of Tongji Medical College, Huazhong University of Science and Technology, Wuhan, Hubei Province, China; 4 Department of Cardiology, Union Hospital of Tongji Medical College, Huazhong University of Science and Technology, Wuhan, Hubei Province, China; French National Center for Scientific Research - Institut de biologie moléculaire et cellulaire, France

## Abstract

Heparanase (HPA), an endo-h-D-glucuronidase that cleaves the heparan sulfate chain of heparan sulfate proteoglycans, is overexpressed in majority of human cancers. Recent evidence suggests that small interfering RNA (siRNA) induces transcriptional gene silencing (TGS) in human cells. In this study, transfection of siRNA against −9/+10 bp (siH3), but not −174/−155 bp (siH1) or −134/−115 bp (siH2) region relative to transcription start site (TSS) locating at 101 bp upstream of the translation start site, resulted in TGS of heparanase in human prostate cancer, bladder cancer, and gastric cancer cells in a sequence-specific manner. Methylation-specific PCR and bisulfite sequencing revealed no DNA methylation of CpG islands within heparanase promoter in siH3-transfected cells. The TGS of heparanase did not involve changes of epigenetic markers histone H3 lysine 9 dimethylation (H3K9me2), histone H3 lysine 27 trimethylation (H3K27me3) or active chromatin marker acetylated histone H3 (AcH3). The regulation of alternative splicing was not involved in siH3-mediated TGS. Instead, siH3 interfered with transcription initiation via decreasing the binding of both RNA polymerase II and transcription factor II B (TFIIB), but not the binding of transcription factors Sp1 or early growth response 1, on the heparanase promoter. Moreover, Argonaute 1 and Argonaute 2 facilitated the decreased binding of RNA polymerase II and TFIIB on heparanase promoter, and were necessary in siH3-induced TGS of heparanase. Stable transfection of the short hairpin RNA construct targeting heparanase TSS (−9/+10 bp) into cancer cells, resulted in decreased proliferation, invasion, metastasis and angiogenesis of cancer cells *in vitro* and in athymic mice models. These results suggest that small RNAs targeting TSS can induce TGS of heparanase via interference with transcription initiation, and significantly suppress the tumor growth, invasion, metastasis and angiogenesis of cancer cells.

## Introduction

Heparanase is an endo-h-D-glucuronidase that has the ability to cleave the heparan sulfate chain of heparan sulfate proteoglycans [1], and facilitates the invasion and metastasis of tumor cells by deteriorating the basement membrane (BM) and extracellular matrix barriers [2]. Heparanase also contributes to angiogenesis by releasing and activating various heparan sulfate-binding growth factors [3], [4]. Moreover, high expression of heparanase is frequently observed in an increasing number of primary human tumors, such as prostate cancer, bladder cancer and gastric cancer, and the heparanase-facilitated invasion and metastasis induce poor outcomes in cancer patients [5]–[8]. These studies suggest that heparanase may be served as a molecular target for cancer therapy.

Silencing of gene expression using small interfering RNA (siRNA) represents a potential strategy for therapeutic product development [9]. In addition to posttranscriptional gene silencing in a wide variety of organisms, siRNA can interact with DNA methyltransferase 3A (DNMT3A) and direct transcriptional gene silencing (TGS) in human cells [10]. Promoter-targeted siRNAs induce the CpG island methylation of ubiquitin C gene [11], human immunodeficiency virus type 1 long terminal repeat [12], Ras association domain family 1A [13], and interleukin-2 [14] in human cells. In addition, exogenous siRNAs trigger TGS in human cells through heterochromatin formation at target promoter, involving recruitment of chromatin-modifying enzymes to result in dimethylation of histone H3 at lysine 9, trimethylation of histone H3 at lysine 27, and histone deacetylation [10], [11], [12]. Moreover, siRNAs targeting intronic or exonic sequences close to an alternative exon can increase the dimethylation of histone H3 at lysine 9 and trimethylation of histone H3 at lysine 27 at the target site, resulting in differential splicing of that exon [15]. These studies suggest that siRNAs affect not only transcription but also splicing process of target gene, implying a feasible approach to develop gene-specific therapeutics.

Transcription start sites (TSS) are essential switches for converting recognition of DNA genome into active synthesis of RNA copies [16]. Vlodavsky *et al*. identified the TSS of heparanase gene at the nucleotide position 99 bp upstream of the translation start site (ATG) by a traditional rapid amplification of cDNA ends (RACE) assay [17]. By using the modified RLM-RACE method to selectively amplify the DNA fragment from capped full-length mRNA, Jiang *et al*. reported the accurate TSS at 101 bp upstream of the ATG [18]. Alternative larger heparanase transcript has also been noted in human immune system, however, the exact location and upstream promoter of its TSS remain largely unknown [19]–[21]. In the current study, we showed that small RNAs targeting heparanase TSS locating at 101 bp upstream of the translation start site [18], including siRNA and short hairpin RNA (shRNA), induced TGS of heparanase via interfering with transcription initiation, but not through inducing heterochromatin formation or epigenetic changes, and attenuated the proliferation, invasion, metastasis and angiogenesis of different kinds of cancer cells *in vitro* and *in vivo*.

## Results

### siRNA-induced TGS of heparanase

**Figure 1:**
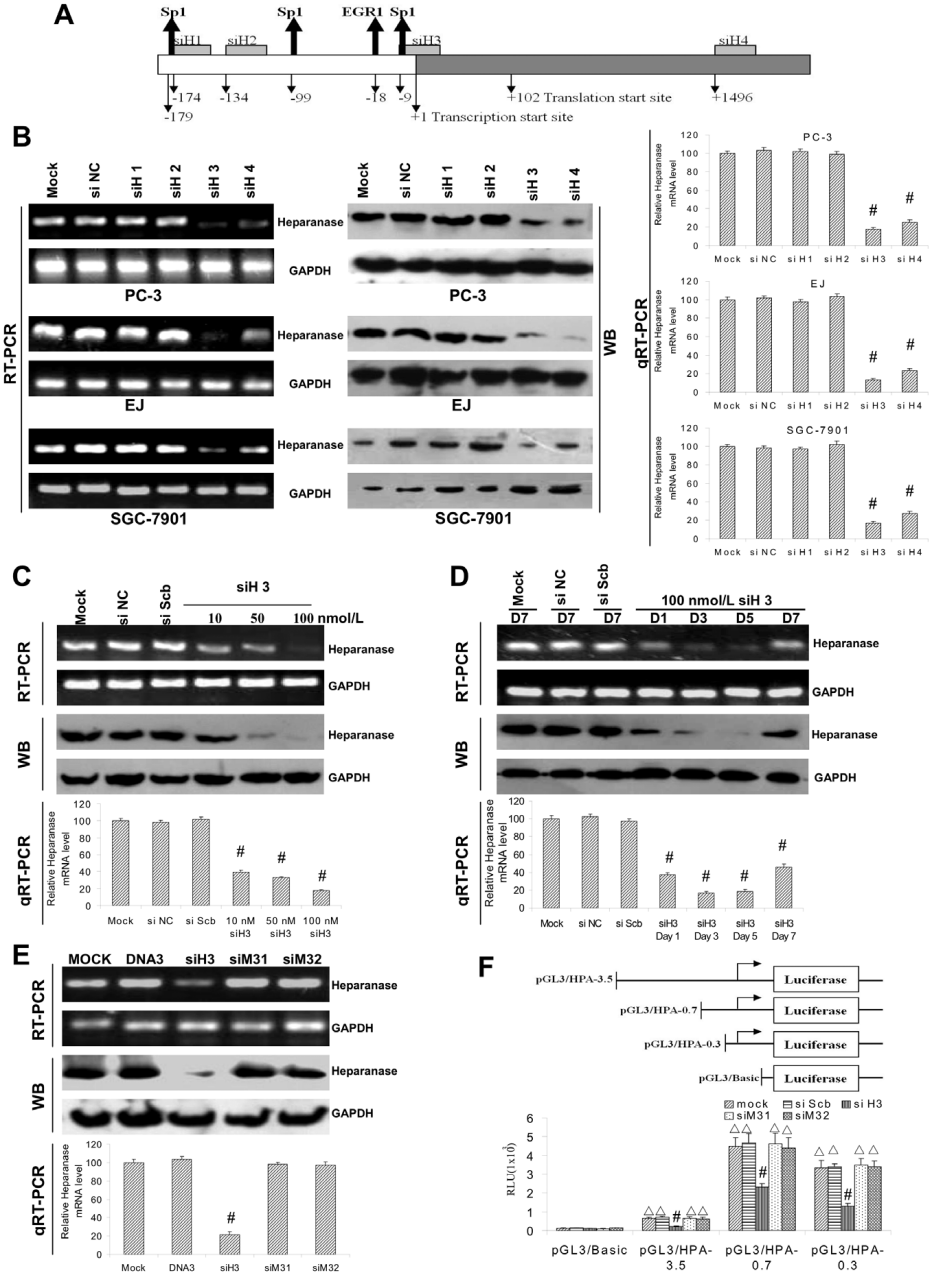
TSS-targeted siRNA abolishes the heparanase expression in cancer cells. **A**, scheme of siRNAs targeting TSS (locating at 101 bp upstream of the translation start site), upstream promoter and encoding regions of heparanase, including siH1 (−174/−155 bp), siH2 (−134/−115 bp), siH3 (−9/+10 bp) and siH4 (+1496/+1515 bp). Automated nucleotide basic local alignment search tool (BLAST) searches demonstrated that there was no sequence similar to siH1, siH2, or siH3 in the transcribed sequence of heparanase. **B**, 72 hrs post-transfection of siRNAs into PC-3, EJ and SGC-7901 cells, 100 nmol/L siH3 or siH4, but not of siH1, siH2 or siNC, attenuated the heparanase expression in cancer cells. **C**, 72 hrs post-transfection of siH3, but not of siNC or siScb, resulted in abolished heparanase expression of cancer cells in a dose-dependent manner. **D**, siH3 (100 nmol/L)-induced gene silencing of heparanase in cancer cells lasted up to 5 days, and partially restored at day 7. **E**, 72 hrs post-transfection of 100 nmol/L DNA duplexes analogous to siH3, siM31 or siM32 (two mismatched siRNAs of siH3), did not abolish the heparanase expression in cancer cells. **F**, dual-luciferase reporter assay indicated that 72 hrs post-transfection of siH3, but not of siScb, siM31 or siM32, suppressed the heparanase promoter activity and transcription in cancer cells. The symbols (# and Δ) indicate a significant decrease from untransfected control (mock) and a significant increase from pGL3/Basic, respectively.

To examine whether siRNA could induce TGS of heparanase, we designed and synthesized three siRNAs targeting TSS (locating at 101 bp upstream of the ATG) or more upstream regions of heparanase promoter, −174/−155 (siH1), −134/−115 (siH2), and −9/+10 bp (siH3) (Fig. 1A and Fig. S1). The siRNA targeting the encoding region +1496/+1515 bp (siH4) was applied as a control (Fig. 1A). Cell lines of most common human cancers, including prostate cancer (PC-3), bladder cancer (EJ) and gastric cancer (SGC-7901), were chosen as models for this study. Transfection of siH3 and siH4, but not of siH1, siH2 or negative control siRNA (siNC), attenuated the heparanase expression in PC-3, EJ and SGC-7901 cells (Fig. 1B). In addition, transfection of siH3, but not of siNC or scrambled siRNA (siSCb), abolished the transcriptional and translational levels of heparanase in a dose-dependent manner (Fig. 1C). The siH3-induced silencing of heparanase lasted up to 5 days, and partially recovered at day 7 (Fig. 1D). Transfection of siM31 and siM32, two mismatched siRNAs of siH3, did not abolish the heparanase expression (Fig. 1E), demonstrating that the interference was executed by siH3 in a sequence-dependent manner. The failure of siM32 to inhibit transcription also indicated that partial complementarity to the 5′-terminus of mRNA was not sufficient for heparanase silencing (Fig. 1E). DNA duplexes analogous to siH3 did not inhibit expression, indicating that recognition must be mediated by RNA (Fig. 1E). Promoter reporter-luciferase assay showed that transfection of siH3, but not siScb, siM31 or siM32, suppressed the heparanase promoter activity and transcription (Fig. 1F), suggesting that the reduced heparanase expression was due to transcriptional inhibition by siH3. Moreover, the expression of non-downstream genes of heparanase, proliferating cell nuclear antigen (PCNA) and cyclin D1, was not affected by transfection of siH3 (Fig. S2). These results indicated that siH3 selectively targeted the TSS and induced TGS of heparanase in a sequence-specific manner in human cancer cells.

### siRNA-induced inhibition of transcription initiation of heparanase

**Figure 2:**
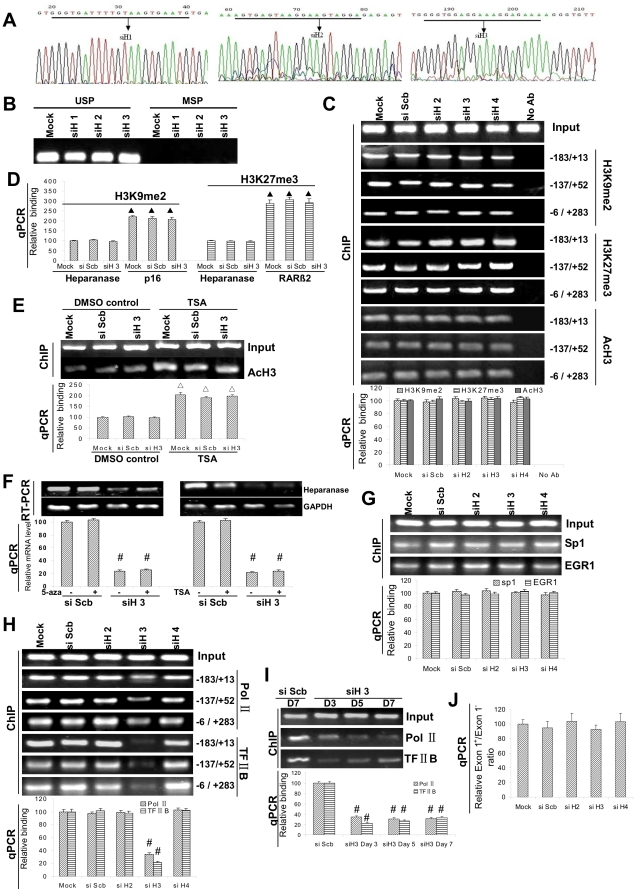
siRNA induces transcription initiation arrest, but not DNA methylation or histone deacetylation, of heparanase promoter. **A** and **B**, bisulfite sequencing and MSP revealed that transfection of siRNAs (100 nmol/L), siH1, siH2 and siH3, did not induce the methylation of CpG islands of heparanase promoter in cancer cells. **C**, 72 hrs post-transfection, ChIP with distinct primer sets indicated that there were no PCR products for “no-antibody” (No Ab) control, and the binding of H3K9me2, H3K27me3 and AcH3 on the heparanase promoter did not change after transfection of siH2, siH3, siH4 or siScb in cancer cells. **D**, The binding of H3K9me2 and H3K27me3 was enriched on the promoter of p16 and RARβ2 compared to that on heparanase promoter in PC-3 cells, respectively. **E,** treatment of siH3- or siScb-transfected cancer cells with TSA (200 nmol/L), resulted in a significant increase in the binding of AcH3 on the heparanase promoter, when compared to those treated with DMSO solvent control. **F**, cancer cells were transfected with siRNAs and treated with 5-Aza-CdR (5 µmol/L) or TSA (200 nmol/L), resulting in no changes in siH3-induced heparanase silencing. **G**, 72 hrs post-transfection, ChIP assay indicated that the binding of Sp1 and EGR1 on the heparanase promoter, did not change after transfection of siH2, siH3, siH4 or siScb in cancer cells. **H**, ChIP assay with distinct primer sets indicated the decreased binding of RNA Pol II and TFIIB on heparanase promoter in siH3-transfected cancer cells, but not in siH2- or siH4-transfected cells. **I**, ChIP assay indicated that the decreased RNA Pol II or TFIIB binding was seen reproducibly and up to 7 days post-transfection of siH3 in cancer cells. **J**, qRT-PCR detection demonstrated that the relative inclusion-to-exclusion ratio of the first exon of heparanase did not change after transfection of siH2, siH3, siH4 or siScb in cancer cells. The symbol (#) indicates a significant decrease from untransfected control (mock) or siScb. The symbol (▴) indicates a significant increase from the binding on heparanase promoter. The symbol (Δ) indicates a significant increase from DMSO control.

Since TGS is associated with epigenetic changes of promoter, such as DNA methylation, histone methylation, and histone deacetylation [10], [11], [12], we first analyzed the epigenetic status of upstream regions of heparanase promoter. The CpG islands were unmethylated in untransfected PC-3, EJ and SGC-7901 cells (mock) and in those transfected with siNC (data not shown). Transfection of siH1, siH2 or siH3, did not induce the methylation of CpG islands of heparanase promoter in cancer cells (Fig. 2A and Fig. 2B). The expression and DNA binding of histone H3 lysine 9 dimethylation (H3K9me2) and histone H3 lysine 27 trimethylation (H3K27me3) on the heparanase promoter, two repressive epigenetic marks involved in TGS [10], did not change upon transfection of siH2, siH3, siH4 or siScb (Fig. S3 and Fig. 2C). Moreover, the binding of acetylated histone H3 (AcH3), a marker of transcriptionally active chromatin [22], was unaffected by transfection with these siRNAs (Fig. 2C). Meanwhile, there were no PCR products for “no-antibody” control in chromatin immunoprecipitation (ChIP) assay (Fig. 2C). The binding of H3K9me2 and H3K27me3 was enriched on the promoter of transcriptionally silenced genes p16 and retinoic acid receptor beta 2 (RARβ2) [23], when compared to that on heparanase promoter in PC-3 cells (Fig. 2D), and the binding of AcH3 on the heparanase promoter significantly increased after the pan histone deacetylases (HDAC) inhibitor trichostatin A (TSA) treatment (Fig. 2E), confirming the adequacy of ChIP assay conditions. In addition, treatment of cancer cells with the DNMT inhibitor 5-aza-2′-deoxycytidine (5-Aza-CdR) or TSA did not affect heparanase silencing, indicating that DNMTs and HDACs were unlikely to be involved in this process (Fig. 2F). Furthermore, ChIP assay revealed no changes in the binding of transcription factors early growth response 1 (EGR1) and Sp1 on the heparanase promoter region surrounding the siH3-targeted site, ruling out their potential roles in the siH3-induced TGS of heparanase (Fig. 2G). However, the decreased binding of RNA polymerase II (RNA Pol II) on heparanase promoter was noted in siH3-transfected cells, but not in siH2-, siH4- or siScb-transfected cells (Fig. 2H). Moreover, the binding of transcription factor II B (TFIIB) that directs RNA Pol II to the core promoter, was also decreased in siH3-transfected cells (Fig. 2H). ChIP data on RNA Pol II or TFIIB binding were confirmed by real-time quantitative PCR (qPCR) with primers spanning the TSS (Fig. 2H). Decreased RNA Pol II or TFIIB binding was seen up to 7 days after transfection (Fig. 2I). In addition, the relative inclusion-to-exclusion ratio of the first exon of heparanase, which was an alternative exon near the siH3-targeted site, did not change after transfection of siH2, siH3, siH4 or siScb in cancer cells (Fig. 2J), suggesting that the regulation of alternative splicing was not involved in siH3-mediated TGS. These results demonstrated that transcription initiation of heparanase was interfered by the siH3 targeting TSS.

### Involvement of Ago1 and Ago2 in siH3-induced transcriptional inhibition of heparanase

**Figure 3:**
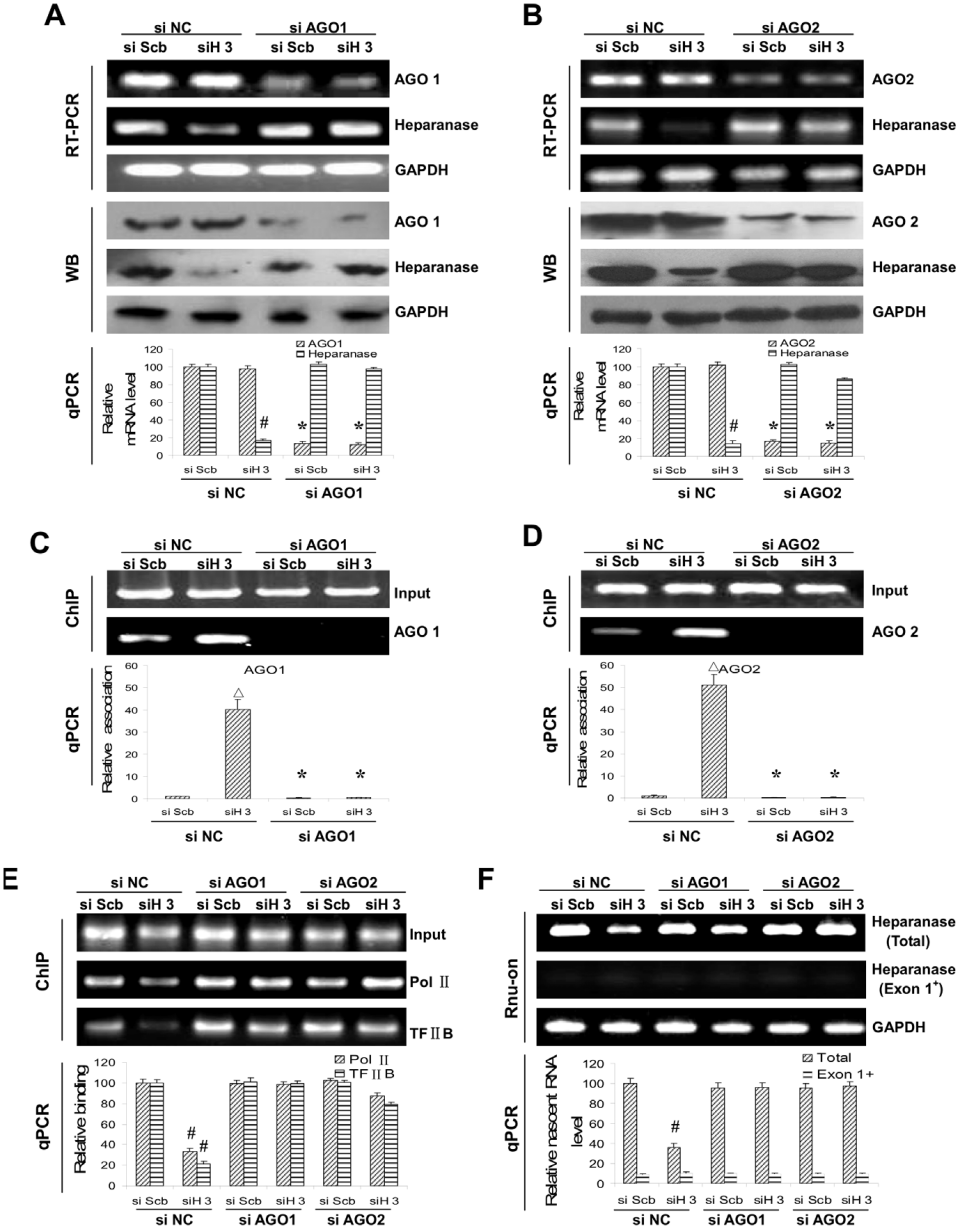
Ago1 and Ago2 are involved in TGS of heparanase induced by TSS-targeted siRNA. **A and B**, knocking down of Ago1 or Ago2 restored the siH3-induced TGS of heparanase in cancer cells, respectively. **C and D**, ChIP assay indicated that 72 hrs post-transfection of siH3, but not of siScb, the association of Ago1 and Ago2 on the heparanase promoter increased in cancer cells, which was abolished by knocking down of Ago1 and Ago2, respectively. **E**, ChIP assay indicated that silencing of Ago1 or Ago2 restored the binding of RNA Pol II and TFIIB on heparanase promoter in cancer cells. **F**, nuclear run-on assay indicated that the upstream transcribed mRNA (containing Exon 1) presented at a low level compared to total heparanase mRNA, and the nascent transcription of total heparanase mRNA, but not the mRNA initiated from upstream alternative TSS, decreased after transfection of siH3 into cancer cells for 72 hrs, which was restored by knocking down of Ago1 or Ago2. The symbols (# and Δ) indicate a significant decrease and a significant increase from siScb, respectively. The symbol (*) indicates a significant decrease from siNC.

Since previous studies indicated the involvement of RNA interference (RNAi) machinery in TGS, we further explored the influence of Argonaute 1 (Ago1) and Argonaute 2 (Ago2), two integral components of the RNAi pathway [24], [25], on siH3-induced TGS of heparanase. As shown in Fig. 3A and Fig. 3B, knocking down of Ago1 or Ago2 suppressed the siH3-induced TGS of heparanase. Moreover, transfection of siH3, but not of siScb, increased the association of Ago1 and Ago2 on the heparanase promoter, which was respectively abolished by knocking down of Ago1 and Ago2 (Fig. 3C and Fig. 3D). In addition, Ago1 or Ago2 silencing increased the binding of RNA Pol II and TFIIB on heparanase promoter, whereas transfection of siNC did not have any effect (Fig. 3E). Nuclear run-on assay further demonstrated that the upstream transcribed mRNA (containing Exon 1) presented at a low level compared to total heparanase mRNA, and transfection of siH3 attenuated the nascent transcription of total heparanase mRNA, but not the mRNA initiated from upstream alternative TSS, which was also restored by knocking down of Ago1 or Ago2, indicating that the reduced heparanase expression was due to inhibition on the transcription initiated from the downstream TSS (Fig. 3F). These results indicated that Ago1 and Ago2 were necessary for siH3-induced transcriptional inhibition of heparanase in human cancer cells.

### RNAi machinery interacted indirectly with transcription preinitiation complex in siH3-induced TGS of heparanase

**Figure 4:**
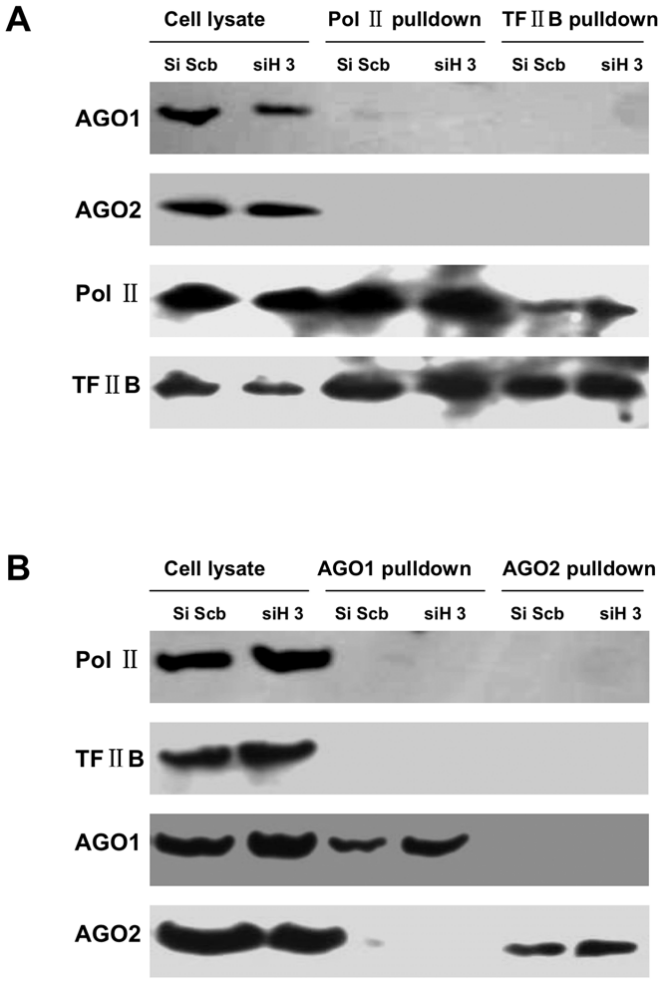
RNAi machinery interacts indirectly with transcription preinitiation complex in siH3-induced TGS of heparanase. **A**, co-immunoprecipitation analysis with pull-down by anti-RNA Pol II or anti-TFIIB antibodies indicated that siH3 or siScb did not attenuate the interaction between RNA Pol II and TFIIB in cancer cells. In addition, Ago1 or Ago2 did not directly interact with either RNA Pol II or TFIIB in siScb- and siH3-transfected cancer cells. **B**, co-immunoprecipitation analysis with pull-down by anti-Ago1 or anti-Ago2 antibodies did not co-immunoprecipite RNA Pol II or TFIIB from siScb- and siH3-transfected cancer cells.

Since recent evidence shows an intricate interaction between the RNAi machinery and RNA Pol II in heterochromatic silencing in *Drosophila* [26], we hypothesize that Ago1 or Ago2 may directly interact with RNA Pol II or TFIIB to participate in siH3-induced TGS of heparanase. Consistent with previous studies [27], [28], co-immunoprecipitation analysis indicated that RNA Pol II interacted with TFIIB in cultured cells. Transfection of siH3 did not attenuate the interaction between RNA Pol II and TFIIB (Fig. 4A). However, anti-RNA Pol II or anti-TFIIB antibodies did not co-immunoprecipite Ago1 or Ago2 from siH3-transfected cells (Fig. 4A), which was further evidenced by co-immunoprecipitation analysis with pull-down by anti-Ago1 or anti-Ago2 antibodies (Fig. 4B). Combining with above evidence that Ago1 and Ago2 influenced the binding of RNA Pol II and TFIIB on heparanase promoter, these results indicated that RNAi machinery and transcription preinitiation complex were associated, but not directly interactive, in siH3-induced TGS of heparanase in human cancer cells.

### Heparanase TSS-targeted shRNA attenuated the proliferation, adhesion, invasion and angiogenesis of cancer cells *in vitro*

**Figure 5:**
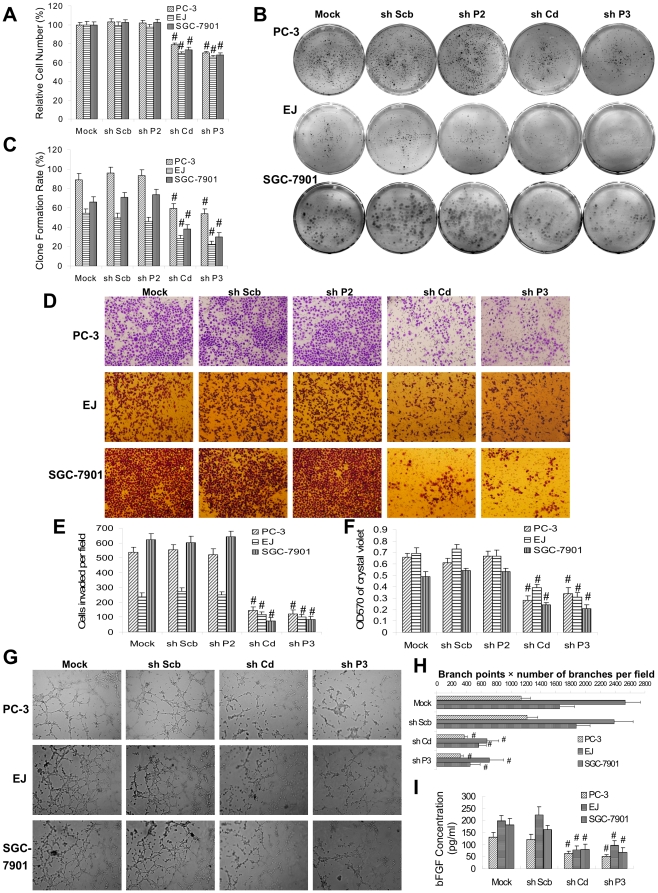
Stable transfection of heparanase TSS-targeted shRNA attenuates the proliferation, adhesion, invasion and angiogenesis of cancer cells *in vitro*. **A**, MTT colorimetry indicated that stable transfection of shP3 or shCd, but not of shP2 or shScb, attenuated the proliferation of PC-3, EJ and SGC-7901 cells. **B** and **C**, colony formation assay indicated that transfection of shP3 and shCd, but not of shP2 or shScb, attenuated the *in vitro* proliferation of PC-3, EJ and SGC-7901 cells. **D** and **E**, transwell analysis indicated that the PC-3, EJ and SGC-7901 cells transfected with shP3 or shCd, but not with shP2 or shScb, possessed an impaired invasion capacity. **F**, in the adhesion assay, PC-3, EJ and SGC-7901 cells transfected with shP3 or shCd, but not with shP2 or shScb, exhibited markedly reduced ability in adhesion to the precoated matrigel. **G** and **H**, endothelial cells were treated with the medium preconditioned by stable transfection of PC-3, EJ and SGC-7901 cells with shP3 or shCd, but not with shScb, resulting in suppressed tube formation on matrigel. **I**, the release of bFGF from PC-3, EJ and SGC-7901 cells was attenuated after transfection of shP3 or shCd, but not of shScb. The symbol (#) indicates a significant decrease from empty vector-transfected cells (mock).

Because recent studies indicate the feasibility of shRNA-mediated TGS in mammalian cells [29], [30], in order to further investigate the effects of heparanase TSS-targeted siRNA on cancer cells, the shRNA constructs were established and transfected into PC-3, EJ and SGC-7901 cells (Fig. S4A). Stable transfection of shP3 (−9/+10 bp) and shCd (+1496/+1515 bp), but not of shP2 (−134/−115 bp) or scrambled shRNA (shScb), resulted in attenuated mRNA and protein levels of heparanase (Fig. S4B, and Fig. S4C), and decreased *in vitro* proliferation of cancer cells (Fig. 5A, Fig. 5B and Fig. 5C). Transwell analysis showed that the cells transfected with shP3 or shCd, but not with shP2 or shScb, presented an impaired invasion capacity (Fig. 5D and Fig. 5E). In addition, cancer cells transfected with shP3 or shCd, but not with shP2 or shScb, exhibited markedly reduced abilities in adhesion to the precoated matrigel (Fig. 5F). The tube formation of endothelial cells was suppressed by treatment with the medium preconditioned by stable transfection of cancer cells with shP3 or shCd, but not with shScb (Fig. 5G and Fig. 5H). Moreover, the release of basic fibroblast growth factor (bFGF) from cancer cells was attenuated after stable transfection of shP3 or shCd, but not of shScb (Fig. 5I). These results indicated that stable transfection of heparanase TSS-targeted shRNA remarkably decreased the proliferation, adhesion, invasion and angiogenesis of cancer cells *in vitro*.

### Heparanase TSS-targeted shRNA inhibited the growth, metastasis and angiogenesis of cancer cells *in vivo*

**Figure 6:**
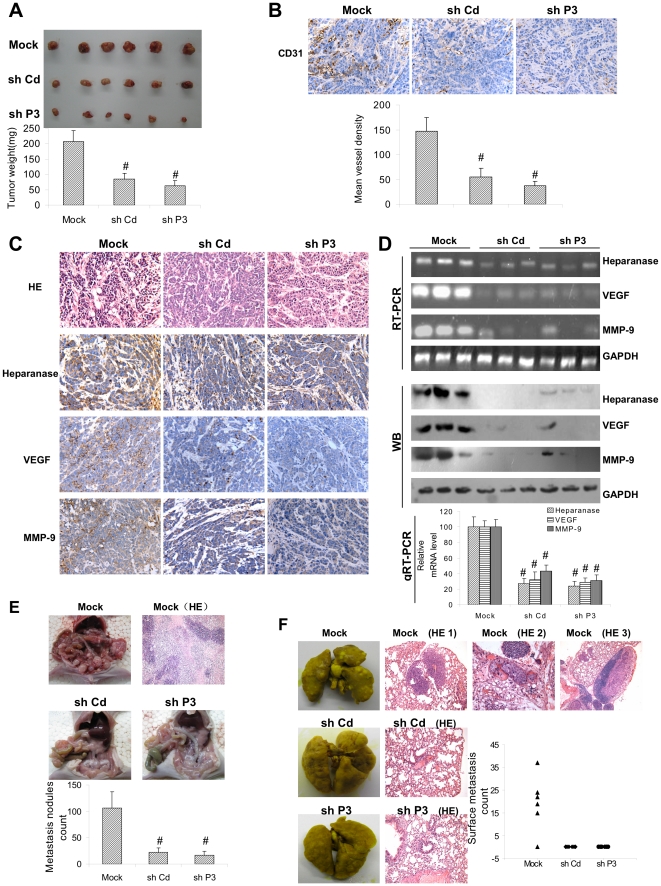
Heparanase TSS-targeted shRNA inhibits the growth, angiogenesis and metastasis of cancer cells *in vivo*. **A**, hypodermic injection of PC-3 cells in athymic nude mice established subcutaneous xenograft tumors. One month later, mice (n = 6) from each group were sacrificed. Stable transfection of cancer cells with shP3 or shCd resulted in decreased tumor size, and the mean tumor weight formed from shP3- or shCd-transfected cells was significantly decreased. **B**, CD31 expression and the mean vessel density within tumors decreased after stable transfection of shP3 or shCd. **C**, HE and immunohistochemical staining revealed that stable transfection of shP3 or shCd resulted in decreased expression of heparanase, VEGF and MMP-9 within tumors. **D**, the expression of heparanase downstream genes within tumors, VEGF and MMP-9, was reduced by stable transfection of shP3 or shCd. **E**, 3×10^6^ PC-3 cells were injected into the peritoneal cavity of 2-month-old male nude mice (n = 5 for each group). Nude mice that received injection of cancer cells stably transfected with shCd or shP3 showed comparatively fewer nodules. **F**, PC-3 cells were injected into the tail vein of athymic nude mice (0.4×10^6^ cells per mouse, n = 5 for each group). Cancer cells stably transfected with shP3 or shCd established significantly fewer metastatic colonies. The symbol (#) indicates a significant decrease from empty vector (mock).

We next investigated the efficacy of shP3 against tumor growth, metastasis and angiogenesis *in vivo*. Stable transfection of shP3 or shCd into PC-3 cells, resulted in decreased growth and weight of subcutaneous xenograft tumors in athymic nude mice (Fig. 6A). In addition, stable transfection of shP3 or shCd resulted in decrease in CD31-positive microvessels and mean vessel density within tumors (Fig. 6B). Moreover, the expression of heparanase downstream genes within tumors, vascular endothelial growth factor (VEGF) and matrix metallopeptidase 9 (MMP-9), was also reduced by stable transfection of shP3 or shCd (Fig. 6C and Fig. 6D).

In the peritoneal metastasis studies, nude mice that received injection of PC-3 cells stably transfected with shCd or shP3 showed comparatively fewer nodules (22±9 and 17±7, respectively) than empty vector (mock) group (106±31) (Fig. 6E). In the experimental metastasis studies, PC-3 cells stably transfected with shP3 or shCd established statistically fewer lung metastatic colonies than mock group (Fig. 6F). Combined with the similar findings in EJ and SGC-7901 cells (data not shown), these results suggested that heparanase TSS-targeted shRNA could inhibit the growth, metastasis and angiogenesis of cancer cells *in vivo*.

## Discussion

The TGS pathway was initially reported in tobacco plants, as the state of methylation and expression of genes were demonstrated to be affected by RNAs [31]. Recently, RNAs have been reported to mediate TGS in mammalian cells via DNA CpG methylation and heterochromatin formation [10], [11], [12], [32]. The silent-state epigenetic modifications cause the loss of transcription factors recruitment [12], [33]. Meanwhile, siRNAs targeting internal gene regions of human fibronectin 1 can inhibit internal elongation and subsequently affect splice site selection [15]. The human heparanase promoter is consisted of a minimal basic region that spans 300–700 bp proximally to the TSS locating at 101 bp upstream of ATG, which is characterized by high GC contents, observed/expected CpG ratio, and the binding sites for several groups of transcription factors [18]. Transcription factor EGR1 is related to the inducible transcription of heparanase gene in T cells [34], whereas the ubiquitous transcription factor Sp1 is associated with its basal transcription [18]. In this study, we designed three siRNAs targeting the heparanase promoter region containing CpG loci and Sp1 binding site, while the siH3-targeted region (−9/+10 bp surrounding heparanase TSS locating at 101 bp upstream of ATG) was also adjacent to the binding site of EGR1. However, our evidence showed that transfection of these siRNAs induced neither methylation of CpG nor heterochromatin formation on heparanase promoter. ChIP analysis further ruled out the changes in the binding of Sp1 and EGR1 on heparanase promoter, and splice variant analysis ruled out the differential splicing of the closest exon.

In fact, siRNAs targeting promoter regions fail to induce TGS in certain instance [35]. Recent studies show that antigene RNAs (agRNAs) targeting the TSS can block gene transcription of human progesterone receptor (PR) [36], [37], androgen receptor (AR) [37], and huntingtin [37], suggesting that TSS in chromosomal DNA provides predictable targets for inhibiting gene expression with RNAs. In the current study, we demonstrated that siRNA and shRNA targeting the TSS induced TGS of heparanase in human cancer cells, while the small RNAs targeting the upstream promoter region containing CpG loci did not suppress the expression of heparanase, suggesting that the loci they targeted might not be susceptible to TGS. Moreover, we also noted the similar efficiencies of siH3 and encoding region-targeted siH4 in attenuating the expression and function of heparanase. These results indicate the valuable role of TSS-targeted small RNAs in the regulation of gene expression by TGS, especially when the RNAs targeting upstream promoter region fail to produce a marked effect.

A mechanism rather than genetic and epigenetic regulation underlines the TGS of heparanase induced by TSS-targeted siRNA in human cancer cells. Similarly, no methylation is observed surrounding the TSS of AR and PR [34], and agRNAs-mediated TGS occurs regardless of whether the promoter contains a TATA box [36], [37]. Previous studies indicate that TFIIB binds the core promoter DNA and directs RNA Pol II to the TSS, promoting assembly of the functional transcription preinitiation complex (PIC) [38]. In this study, we showed that siRNAs targeting the TSS locating at 101 bp upstream of ATG interfered with transcription initiation of heparanase, and the concomitant loss of TFIIB and RNA Pol II suggested that the siRNAs reduced the assembly of PIC formation at this TSS. It has been indicated that when mammalian RNA polymerase binds to DNA at TSS, it forms an open complex in which bases −9 to +2 are accessible to chemical agents that modify single-stranded DNA, suggesting that TSS may be accessible to hybridization [36], [37]. Recent studies have indicated the presence of noncoding promoter-associated RNA (pRNA) and the consequences of their targeting by siRNAs in human cells [39], [40]. Low-copy pRNAs are recognized by the antisense strand of the siRNA and function as a recognition motif to direct epigenetic silencing complexes to the corresponding targeted promoters to mediate TGS in human cells [39]. In addition, the formation of siRNA-pRNA complex with the contribution of Ago2 is responsible for the reduced assembly of functional PIC and blockade of transcription initiation in the c-myc promoter [40]. Thus, whether pRNA exists to direct siH3-induced TGS of heparanase warrants our further investigation. Because an open complex is formed during the transcription of every gene, it is feasible to directly design agRNAs to any gene that has a characterized TSS [41]. Therefore, we believe that the TGS strategy via TSS-targeted siRNA may also be applied to other genes, which warrants our further investigation.

Ago protein complexes containing single-stranded small RNAs are called mature RNA-induced silencing complex (RISC) [42]. Ago proteins have positively charged surfaces that are well suited for binding siRNA and aligning it with the complementary target sequence, suggesting that Ago proteins may play a central role in the formation of RNA-induced initiation of TGS complex that initiates the heterochromatin formation [37]. In this study, we demonstrated that Ago1 and Ago2 were necessary to attenuate the binding of RNA Pol II and TFIIB on heparanase promoter during siH3-induced TGS of heparanase. Recent evidence indicates that the interaction between RNA Pol II and the small RNA machinery (Ago1, Ago2, piwi) affects heterochromatic silencing in *Drosophila* [26]. In human cells, Ago1 associates with RNA Pol II via protein-protein interactions and is required for dimethylation of histone H3 at lysine 9 and TGS [32]. In this study, our data did not show any direct protein-protein interaction between the RISC (Ago1 and Ago2) and PIC (RNA Pol II and TFIIB) components during siH3-induced TGS of heparanase, suggesting that different association between RISC and PIC might be involved in variable siRNA-induced TGS.

In experimental models, the cells expressing heparanase possess a high potential for extravasation of tumor cells in vascular vessels and are susceptible to develop a lung metastasis [43]. Apart from its direct involvement in BM invasion by endothelial cells, heparanase elicits an indirect angiogenic response through releasing angiogenic growth factors, such as bFGF and VEGF [3], [4]. Thus, heparanase may facilitate tumor cell invasion and neovascularization, both critical steps in tumor progression. In the current study, we further demonstrated that TSS-targeted shRNA significantly inhibited heparanase expression and suppressed the invasion, metastasis and angiogenesis of cancer cells *in vitro* and *in vivo*. Our findings suggest that induction of TGS by TSS-targeted shRNA is a promising approach for the suppression of heparanase gene function as well as illustrating its application in cancer therapy.

In summary, in this study, we have shown that small RNAs (siRNA or shRNA) targeting the TSS locating at 101 bp upstream of the translation start site can significantly suppress transcription initiation, but not induce epigenetic changes, to mediate TGS of heparanase and attenuate the invasion, metastasis and angiogenesis of human cancer cells *in vitro* and *in vivo*. The consistent findings in cell lines originating from different cancer types imply the generality of this strategy in cancer therapy. Although low levels of alternative upstream transcribed heparanase transcript were also noted in this study, its exact TSS location, 5′-flanking region and promoter sequence still remain exclusive so far. Until these items have been elucidated, the effects of siRNAs targeting this TSS and promoter can be further investigated. We believe that TSS-targeted small RNAs have the potential to be developed into a useful approach for inhibition of metastatic growth of human cancer.

## Materials and Methods

### Cell culture

Human bladder cancer cell line EJ (MGH-U1) was obtained from the Institute of Urology, Peking University (Beijing, China). Human prostate cancer cell line PC-3 (CRL-1435) and endothelial cell line HUVEC (CRL-1730) were purchased from American Type Culture Collection (Rockville, MD). Human gastric cancer cell line SGC-7901 was obtained from the Type Culture Collection of Chinese Academy of Sciences (Shanghai, China). The cells were grown in RPMI1640 medium (Life Technologies, Inc., Gaithersburg, MD) supplemented with 10% fetal bovine serum (FBS, Life Technologies, Inc.), and applied for transcfection or treatment with 5-Aza-CdR (Sigma, St. Louis, MO) or TSA (Sigma) as previously described [44], [45].

### siRNA transfection

Three 21-nucleotide siRNAs targeting TSS (locating at 101 bp upstream of ATG) or more upstream regions of heparanase promoter, siH1 (−174/−155 bp), siH2 (−134/−115 bp) and siH3 (−9/+10 bp), were designed according to the TSS site, CpG loci and transcription factor binding sites within heparanase promoter. These siRNAs were chemically synthesized and transfected with Genesilencer Transfection Reagent (Genlantis, San Diego, CA) according to the suggested concentrations of manufacturer (RiboBio Co. Ltd, Guangzhou, China). The siH4 (+1496/+1515 bp) targeting the encoding region of heparanase served as a positive control [46]. The siNC and siSCb were applied as controls. The untransfected cells were applied as a mock control. For knocking down of Ago1 and Ago2, the cells were co-transfected with the corresponding siRNAs. The nucleotide sequences of these siRNAs were shown in Table S1.

### shRNA constructs and stable transfection

Three sets of oligonucleotides encoding shRNAs complementary to TSS (locating at 101 bp upstream of ATG), upstream promoter and encoding regions of heparanase were subcloned into pGenesil-1 (Genesil Biotechnology, Wuhan, China). Annealed oligonucleotides were cloned downstream of U6 promoter as shown in Table S1. The plasmids shP2 (−134/−115 bp), shP3 (−9/+10 bp), shCd (+1496/+1515 bp) and shScb were verified by DNA sequencing and transfected into cancer cells with Genesilencer Transfection Reagent (Genlantis). Stable cancer cell lines transfected with shRNA were screened by administration of G_418_ (Invitrogen, Carlsbad, CA).

### Dual-luciferase reporter assay for heparanase promoter activity

The human heparanase promoter-luciferase reporter constructs containing a series of deletion fragments from the 5′-flanking region of heparanase promoter, pGL3/HPA-3.5, pGL3/HPA-0.7 and pGL3/HPA-0.3, were kindly provided by Dr. Xiulong Xu (Department of General Surgery, Rush University Medical Center, Chicago, IL) [18]. Dual-luciferase reporter assay was performed according to the manufacturer's instructions (Promega, Madison, WI). The luciferase activity was measured with a luminometer (Lumat LB9507, Berthold Tech., Bad Wildbad, Germany).

### RT-PCR and real-time quantitative RT-PCR

RT-PCR was performed as previously described [46], with PCR primers shown in Table S2. Real-time quantitative RT-PCR (qRT-PCR) with SYBR Green PCR Master Mix (Applied Biosystems, Foster City, CA) was performed using ABI Prism 7700 Sequence Detector (Applied Biosystems). The fluorescent signals were collected during extension phase, Ct values of the samples were calculated, and the transcript levels were analyzed by 2^−ΔΔCt^ method. For splice variant analysis [15], primers were designed to amplify the transcripts containing the first exon of heparanase, and the relative inclusion-to-exclusion ratio of the first exon (Exon 1^+^/Exon 1^−^) was determined by measuring relative abundance of Exon 1 to the remaining total heparanase transcripts that did not contain Exon 1.

### Western blot

Western blot was performed as previously described [46], with antibodies specific for heparanase (InSight Company, Rehovot, Israel), Sp1 (Abcam, Cambridge, MA), H3K9me2, H3K27me3, AcH3, RNA Pol II (Upstate Biotechnology, Temacula, CA), Ago1, Ago2 (Cell Signaling Technology, Inc., Danvers, MA), EGR1, TFIIB, and GAPDH (Santa Cruz Biotechnology, Santa Cruz, CA).

### Methylation analysis of promoter CpG islands

Genomic DNA was extracted with the DNeasy Tissue Kit (Qiagen Inc., Valencia, CA) according to the manufacturer's instructions. Sodium bisulfite modification of genomic DNA was performed as previously described [5], [6]. The methylation-specific PCR (MSP) was undertaken to analyze the methylation of heparanase promoter with primers spanning the CpG islands (Table S1). The PCR products amplified with a pair of universal primers were resorted to direct bisulfite sequencing (TakaRa Bio., Inc., Shiga, Japan).

### Chromatin immunoprecipitation

ChIP assay was performed according to the manufacture's instructions of the EZ-ChIP kit (Upstate Biotechnology). The PCR primers were designed by Premier Primer 5.0 software to amplify three adjacent regions (Table S2), −183/+13, −137/+52 and −6/+283, surrounding the heparanase TSS (locating at 101 bp upstream of ATG). qPCR with SYBR Green PCR Master Mix (Applied Biosystems) was performed using ABI Prism 7700 Sequence Detector (Applied Biosystems). The amount of immunoprecipitated DNA was calculated in reference to a standard curve and normalized to input DNA.

### Nuclear run-on assay

Nuclear run-on assays were performed based on the incorporation of biotin-16-uridine- 5′-triphosphate (biotin-16-UTP) in nascent transcripts as previously described [47]. Briefly, 5×10^6^ nuclei of siRNA-transfected cells were isolated and consequently incubated in a reaction buffer containing rNTPs and biotin-16-UTP (Roche, Indianapolis, IN, USA) at 30°C for 45 min. The reaction was stopped by adding RNase-free DNase I (Sigma), and the nuclei were lysed and treated with proteinase K (Sigma). Total RNA was extracted using Trizol (Invitrogen), and biotinylated nascent RNA was purified using agarose-conjugated streptavidin beads (Invitrogen). Beads were then eluted, and biotinylated RNA was isolated for RT-PCR and qRT-PCR assays.

### Co-immunoprecipitation

Co-immunoprecipitation was performed as previously described [48], with antibodies specific for RNA Pol II, TFIIB, Ago1, Ago2 or unspecific IgG (Santa Cruz Biotechnology). The bead-bound proteins were released by boiling the protein A-Sepharose beads (Santa Cruz Biotechnology) in 1×SDS-PAGE loading buffer and analyzed by western blot.

### Cell viability, colony formation, adhesion and invasion assay

Cell viability was monitored by 2-(4,5-dimethyltriazol-2-yl)-2,5-diphenyl tetrazolium bromide (MTT, Sigma) colorimetric assay [46]. Colony formation ratios and cell adhesion on 96-well plates precoated with matrigel (BD Biosciences, Franklin Lakes, NJ) were measured as previously described [46]. The Boyden chamber technique (transwell analysis) with matrigel-coated filters was performed for cell invasion assay [42].

### In vitro angiogenesis assay

The endothelial tube–like formation assay of HUVEC cells was performed as previously described [46]. The bFGF levels were examined with an enzyme-linked immunosorbent assay kit (Cusabio Biotech Co., Ltd, China).

### In vivo metastasis assay

All animal experiments were approved by the Animal Care Committee of Tongji Medical College (approval number: Y20080290). For the *in vivo* tumor growth studies, 2-month-old male nude mice (n = 6 per group) were injected subcutaneously in the lower back with 1×10^6^ cancer cells stably transfected with shRNAs. One month later, mice were sacrificed and examined for tumor weight, gene expression, and angiogenesis. The peritoneal metastasis (3×10^6^ cancer cells per mouse) and experimental metastasis (0.4×10^6^ cancer cells per mouse) studies were performed with 2-month-old male nude mice as previously described [49], [50].

### Immunohistochemistry

Immunohistochemical staining was performed as previously described [51], with antibodies specific for CD31, MMP-9, VEGF (Santa Cruz Biotechnology; 1∶200 dilutions) or heparanase (InSight Company; 1∶100 dilution).

### Statistical analysis

Unless otherwise stated, all data were shown as mean ± standard error of the mean (SEM). Statistical significance (*P*<0.05) was determined by *t* test or analysis of variance (ANOVA) followed by assessment of difference using SPSS 12.0 software (SPSS Inc., Chicago, IL).

## Supporting Information

Figure S1 **Schematic location of alternative heparanase TSS.** The transcription of heparanase gene can be initiated from two different TSS sites, resulting in two mRNA species, which contain Exon 1 or not, respectively. The location and promoter region of former TSS remain unknown, while the later TSS is located at or nearly close to 101 bp upstream of the translation start site (ATG).
(DOC)

Figure S2 **Target specificity of heparanase TSS-targeted siRNA.** Cancer cells were transfected with 10–100 nmol/L of siH3, siNC (100 nmol/L) and siScb (100 nmol/L) or left untreated. Cells were collected at 72 hrs post-transfection. qRT-PCR indicated that the expression of non-downstream genes of heparanase, PCNA and cyclin D1, was not affected by transfection of siH3, siNC or siScb.
(DOC)

Figure S3 **Heparanase TSS-targeted siRNA does not influence the expression of epigenetic and transcriptionally active chromatin marks.** Cancer cells were transfected with 100 nmol/L of siH3 or siScb for various duration as indicated. Western blot revealed that transfection of siH3 or siScb did not affect the expression of H3K9me2, H3K27me3, AcH3, RNA Pol II, TFIIB, Sp1 or EGR1 in cancer cells.
(DOC)

Figure S4 **Establishment of stable cell lines transfected with heparanase TSS-targeted shRNA.** The shRNA constructs targeting TSS (locating at 101 bp upstream of the translation start site), upstream promoter and encoding regions of heparanase, shP2 (−134/−115 bp), shP3 (−9/+10 bp), shCd (+1496/+1515 bp) and shScb, were transfected into cultured cancer cell lines PC-3, EJ and SGC-7901, respectively. **A**, 72 hrs post-transfection, the transfection efficiency was monitored by the reporter gene, enhanced green fluorescent protein (EGFP), within the vectors. **B**, stable cell lines were established by administration of G_418_. RT-PCR (left panel) and western blot (right panel) demonstrated that stable transfection of shP3 or shCd resulted in attenuated mRNA and protein levels of heparanase in cancer cells. **C**, qRT-PCR further indicated that the heparanase mRNA levels in cancer cells were attenuated by stable transfection of shP3 or shCd. The symbol (#) indicates a significant decrease from vector transfection (mock) group.
(DOC)

Table S1 **Sequences of small interfering RNAs and short hairpin RNAs.**
(DOC)

Table S2 **Primers sets used for RT-PCR, ChIP and qPCR.**
(DOC)
