# Supplementary material for: Small RNAs Targeting Transcription Start Site Induce Heparanase Silencing through Interference with Transcription Initiation in Human Cancer Cells
Source: PLoS One. 2012 Feb 20;7(2):e31379. doi: 10.1371/journal.pone.0031379 (PMC3282686; doi:10.1371/journal.pone.0031379)
Supplement: Figure S2 — Target specificity of heparanase TSS-targeted siRNA. Cancer cells were transfected with 10–100 nmol/L of siH3, siNC (100 nmol/L) and siScb (100 nmol/L) or left untreated. Cells were collected at 72 hrs post-transfection. qRT-PCR indicated that the expression of non-downstream genes of heparanase, PCNA and cyclin D1, was not affected by transfection of siH3, siNC or siScb. (DOC) [file pone.0031379.s002.doc]

**qRT-PCR**

**Supplementary Figure S2**
