# Supplementary figures and images for: Small RNAs Targeting Transcription Start Site Induce Heparanase Silencing through Interference with Transcription Initiation in Human Cancer Cells
Source: PLoS One. 2012 Feb 20;7(2):e31379. doi: 10.1371/journal.pone.0031379 (PMC3282686; doi:10.1371/journal.pone.0031379)

**si Scb siH 3**

**D7 D3 D5 D7**

**H3K9me2**


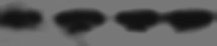


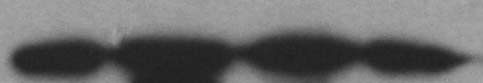


**H3K27me3**


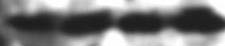


**AcH3**


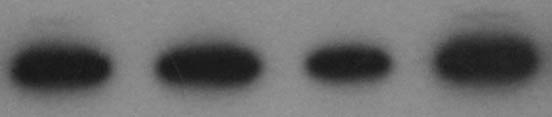


**WB**

**Pol Ⅱ**


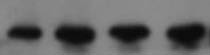


**TFⅡB**


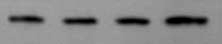


**Sp1**


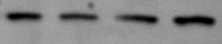


**EGR1**


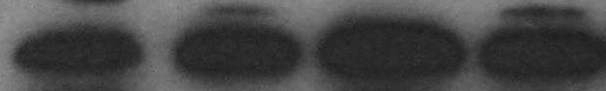


**GAPDH**

**Supplementary Figure S3**

Supplement: Figure S3 — Heparanase TSS-targeted siRNA does not influence the expression of epigenetic and transcriptionally active chromatin marks. Cancer cells were transfected with 100 nmol/L of siH3 or siScb for various duration as indicated. Western blot revealed that transfection of siH3 or siScb did not affect the expression of H3K9me2, H3K27me3, AcH3, RNA Pol II, TFIIB, Sp1 or EGR1 in cancer cells. (DOC) [file pone.0031379.s003.doc]
