# Supplementary material for: Small RNAs Targeting Transcription Start Site Induce Heparanase Silencing through Interference with Transcription Initiation in Human Cancer Cells
Source: PLoS One. 2012 Feb 20;7(2):e31379. doi: 10.1371/journal.pone.0031379 (PMC3282686; doi:10.1371/journal.pone.0031379)
Supplement: Figure S4 — Establishment of stable cell lines transfected with heparanase TSS-targeted shRNA. The shRNA constructs targeting TSS (locating at 101 bp upstream of the translation start site), upstream promoter and encoding regions of heparanase, shP2 (−134/−115 bp), shP3 (−9/+10 bp), shCd (+1496/+1515 bp) and shScb, were transfected into cultured cancer cell lines PC-3, EJ and SGC-7901, respectively. A, 72 hrs post-transfection, the transfection efficiency was monitored by the reporter gene, enhanced green fluorescent protein (EGFP), within the vectors. B, stable cell lines were established by administration of G418. RT-PCR (left panel) and western blot (right panel) demonstrated that stable transfection of shP3 or shCd resulted in attenuated mRNA and protein levels of heparanase in cancer cells. C, qRT-PCR further indicated that the heparanase mRNA levels in cancer cells were attenuated by stable transfection of shP3 or shCd. The symbol (#) indicates a significant decrease from vector transfection (mock) group. (DOC) [file pone.0031379.s004.doc]

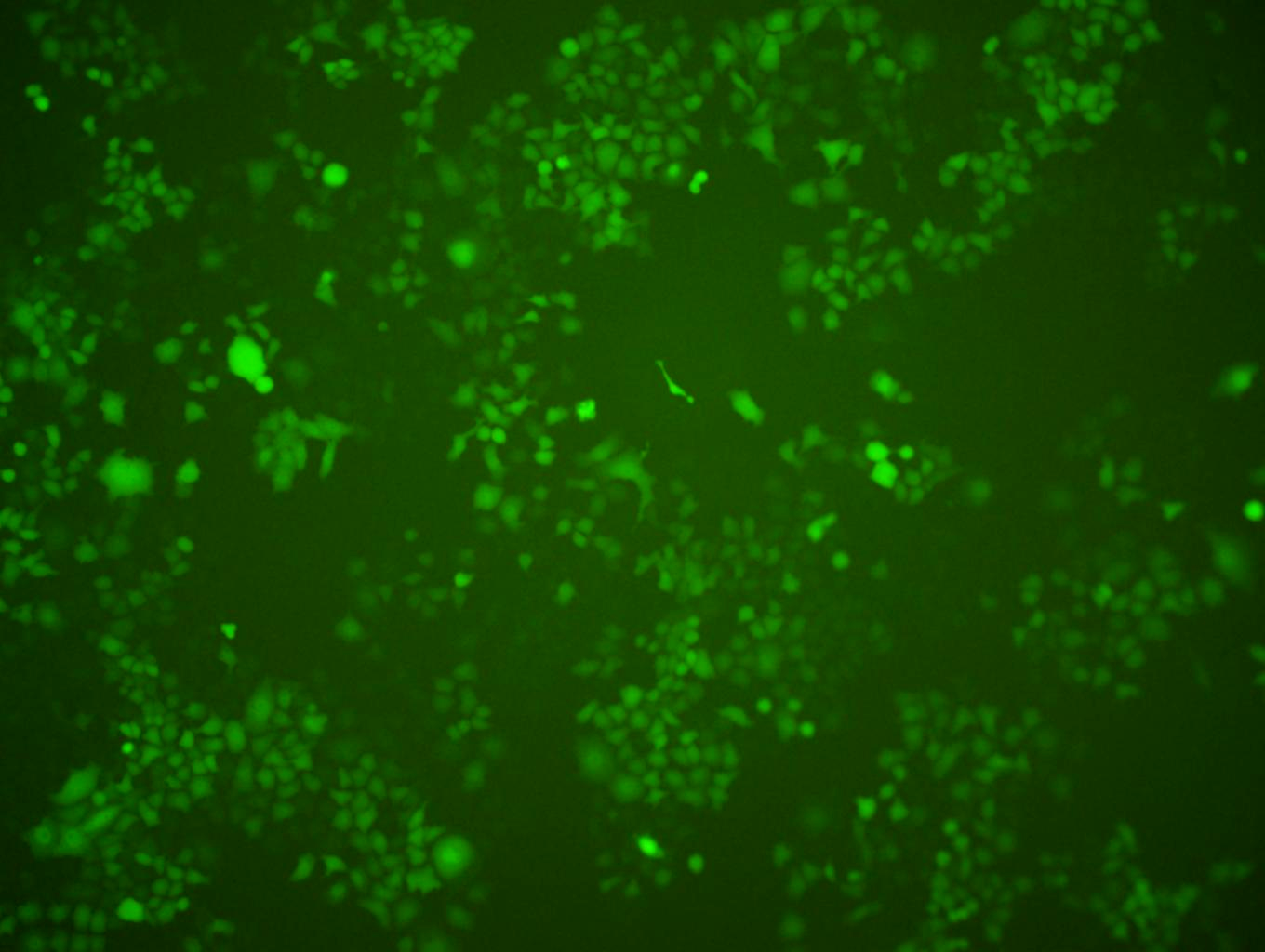

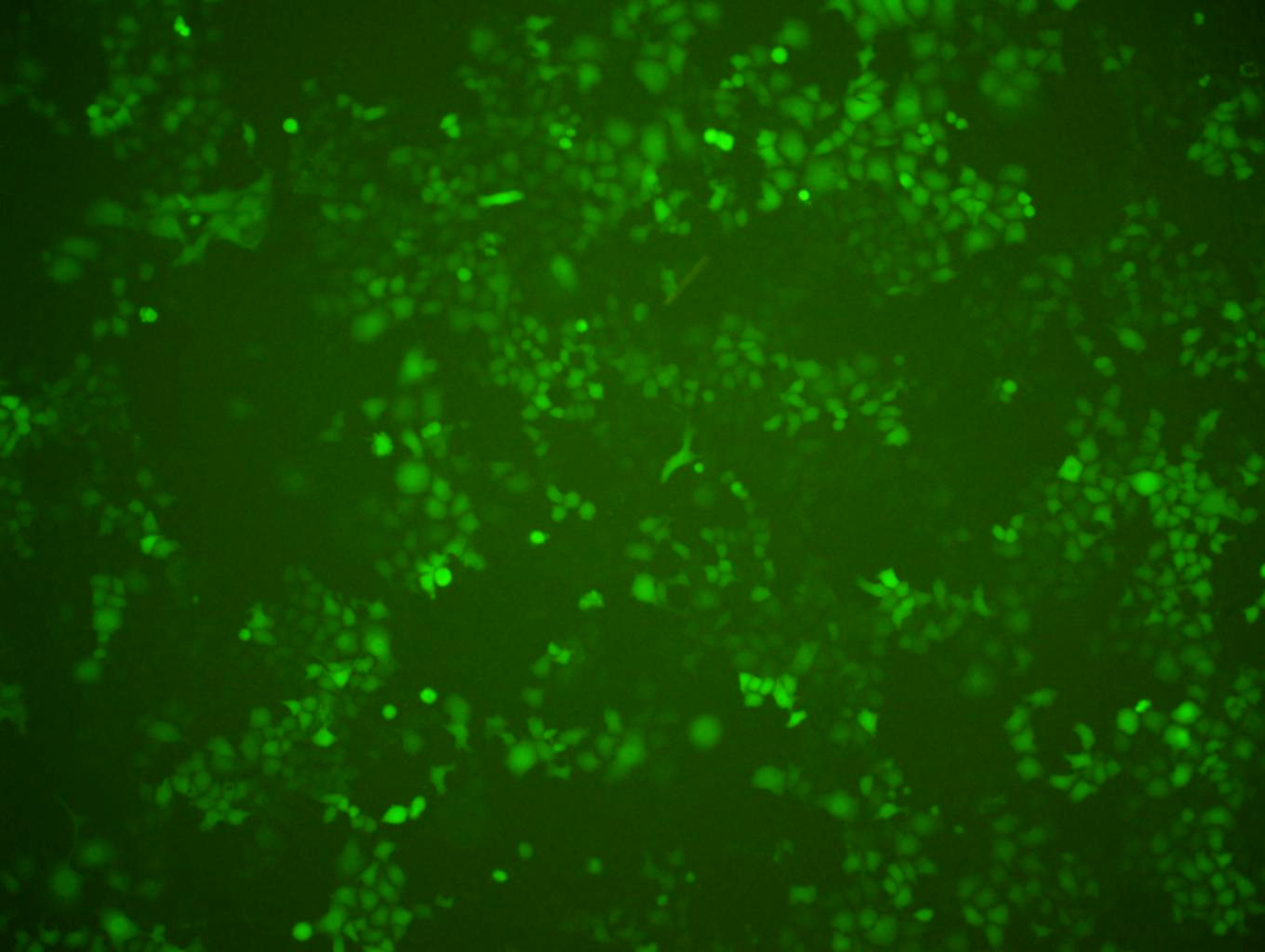

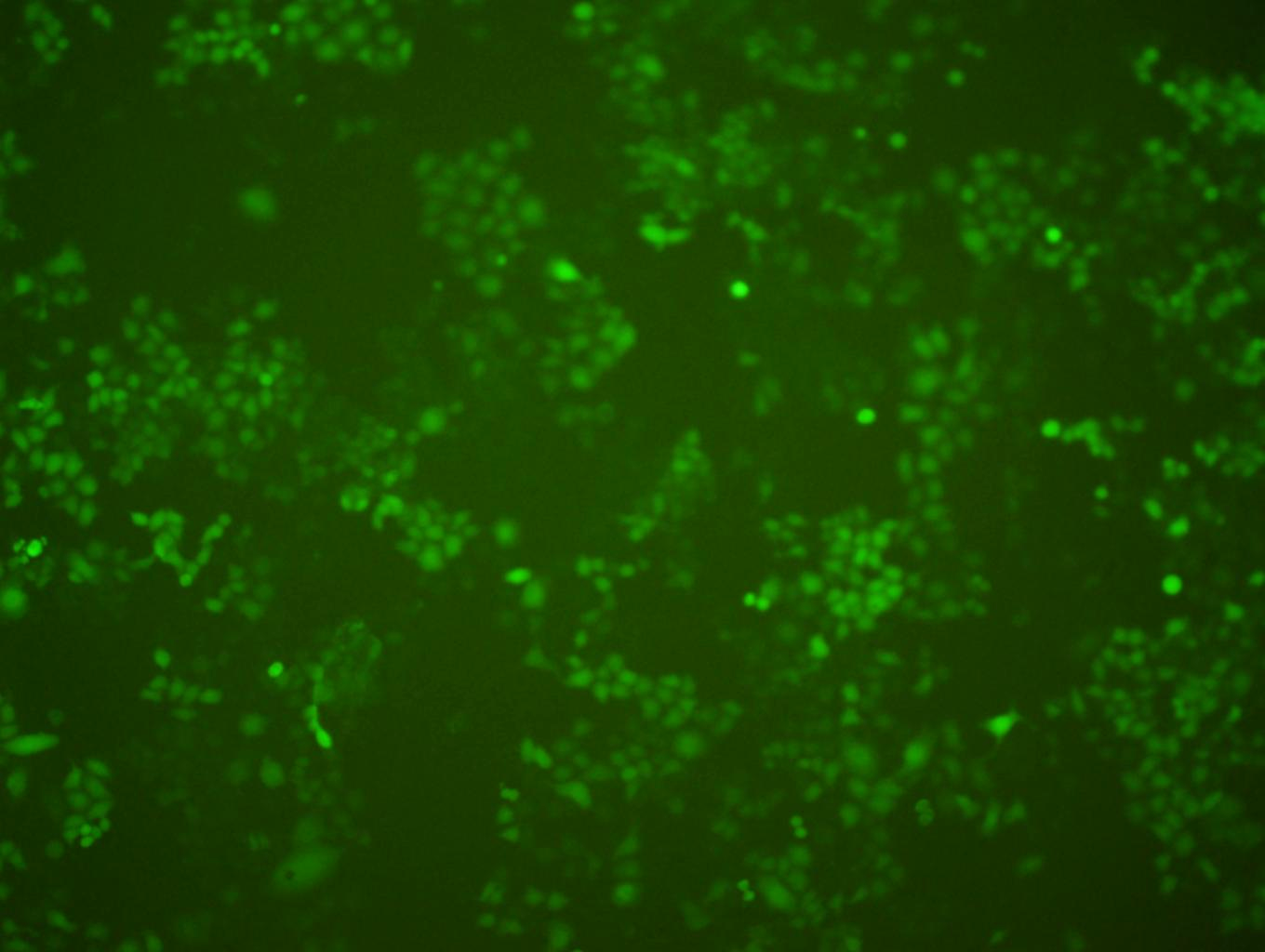

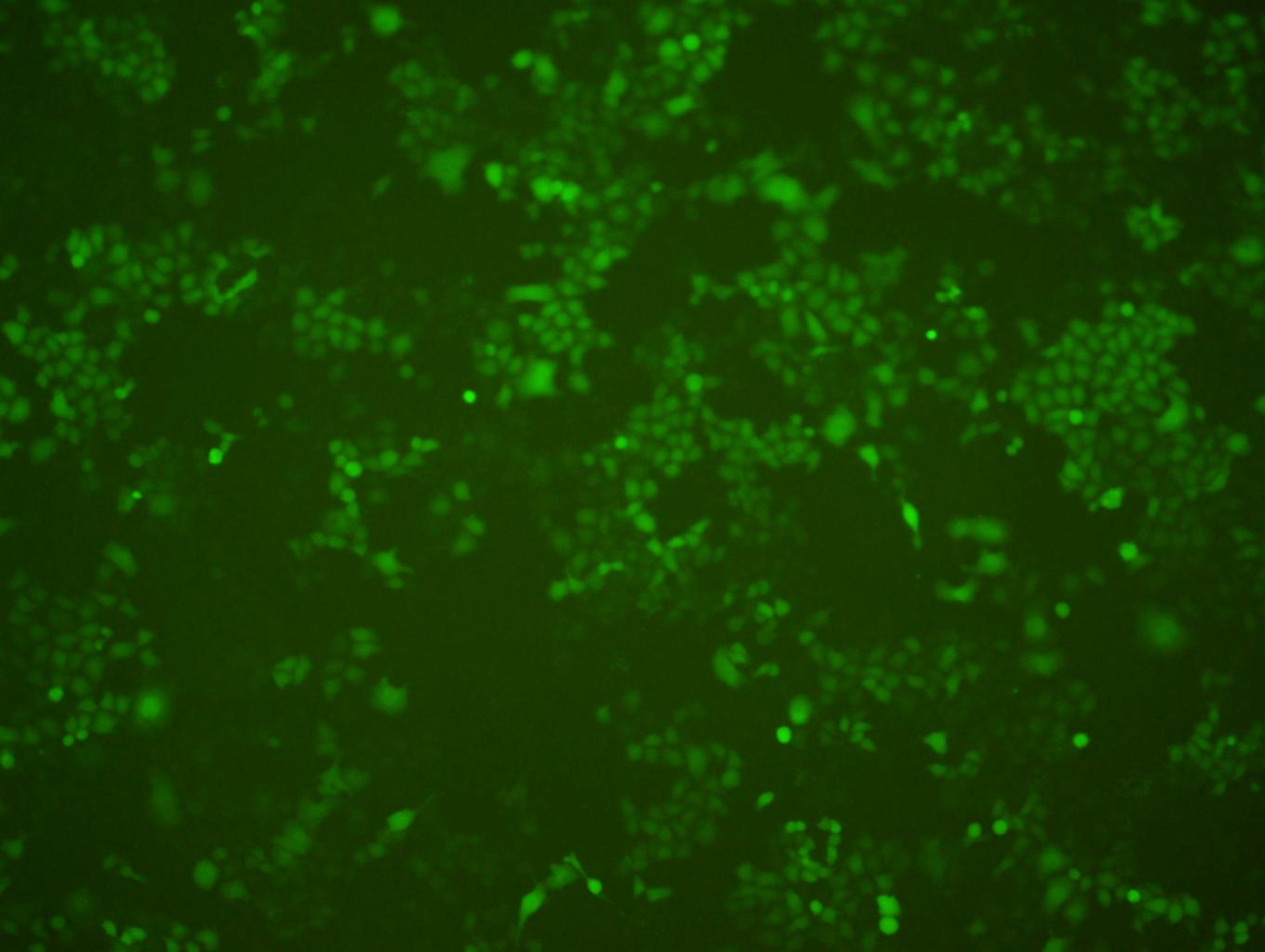

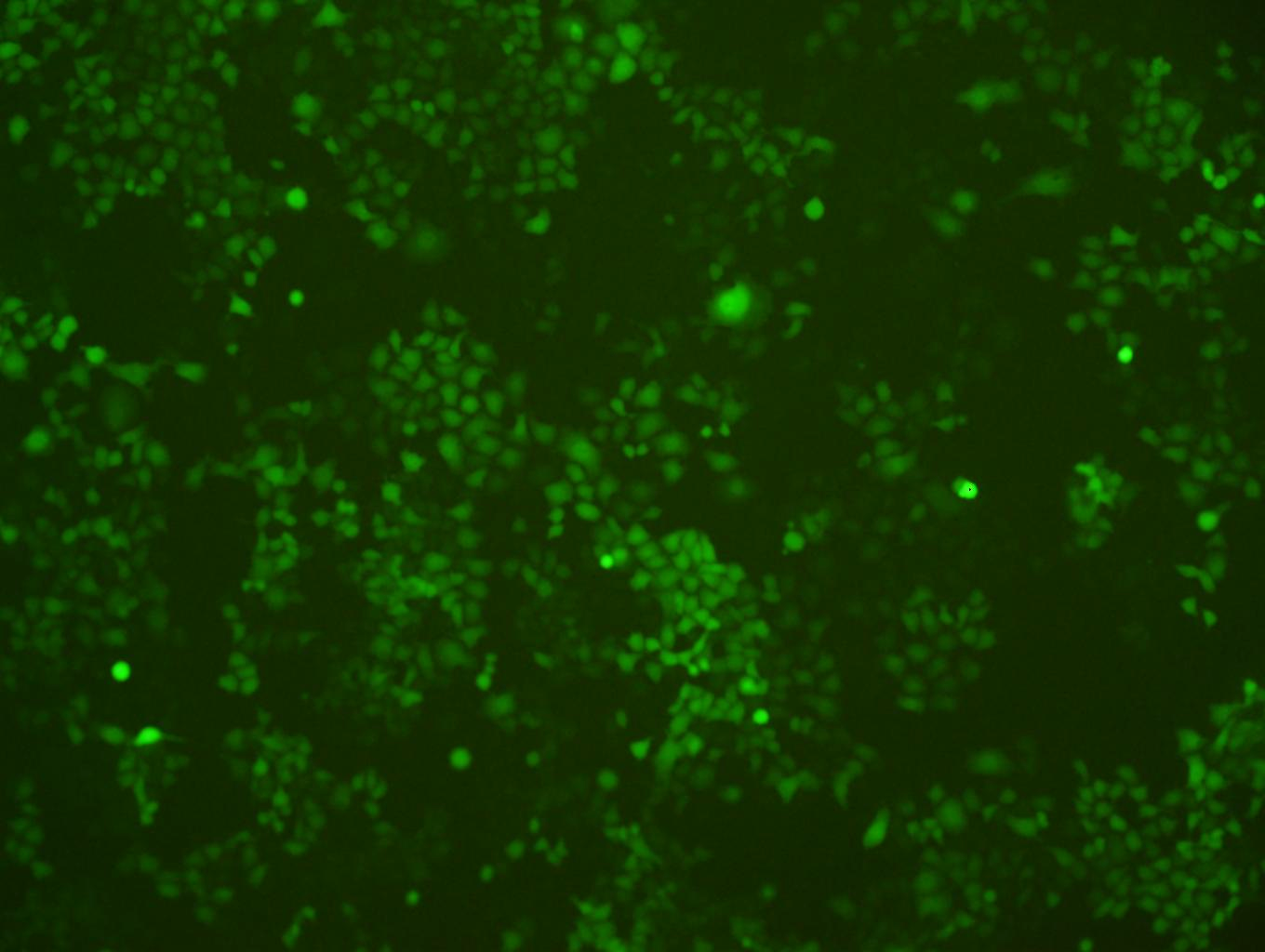


**Mock transfection sh Scb transfection sh P2 transfection sh Cd transfection sh P3 transfection**

**PC-3**

**A**


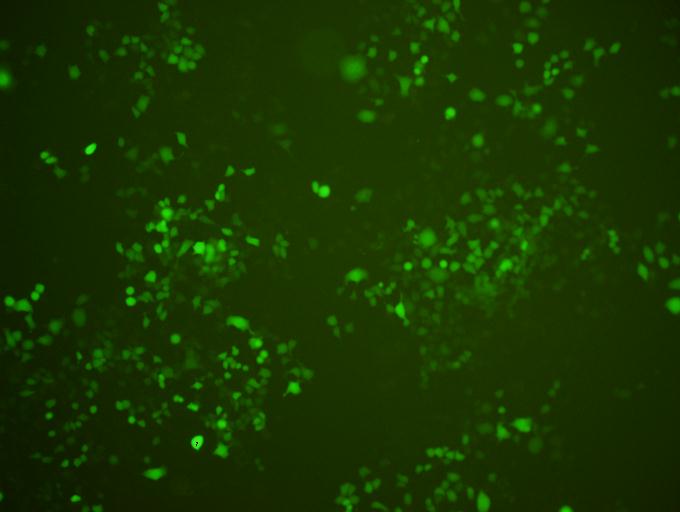

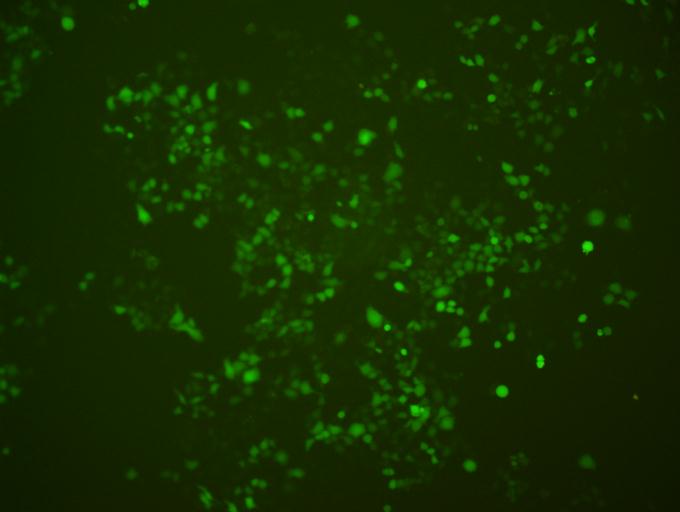

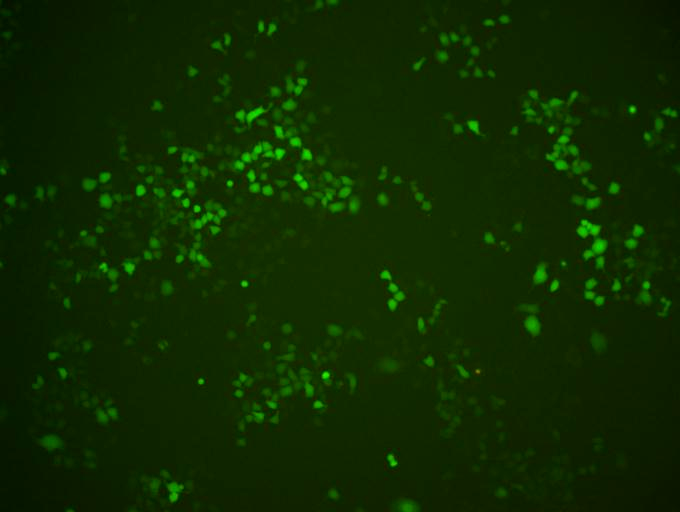

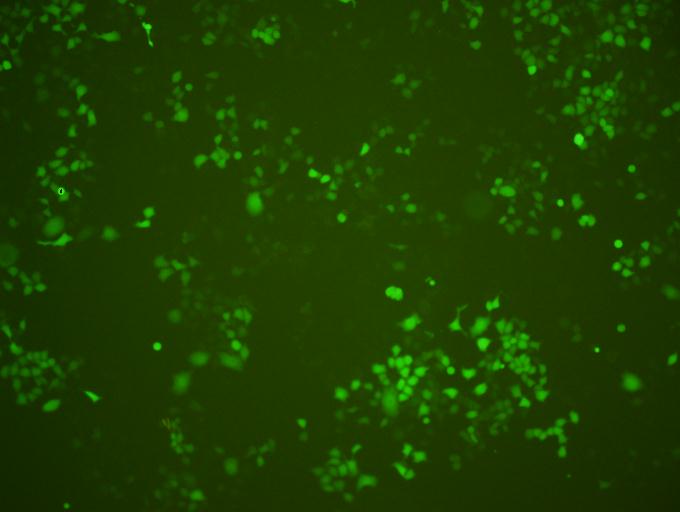

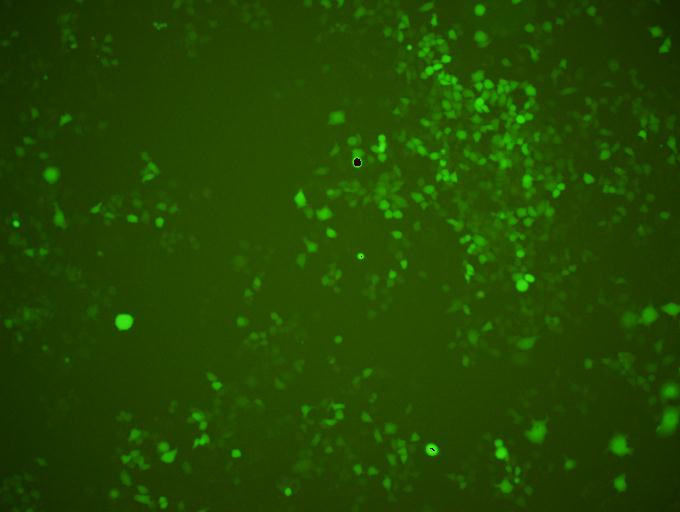


**EJ**


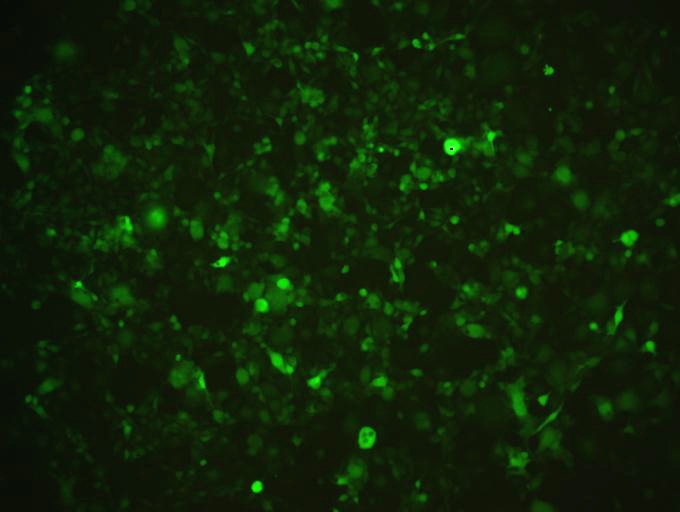

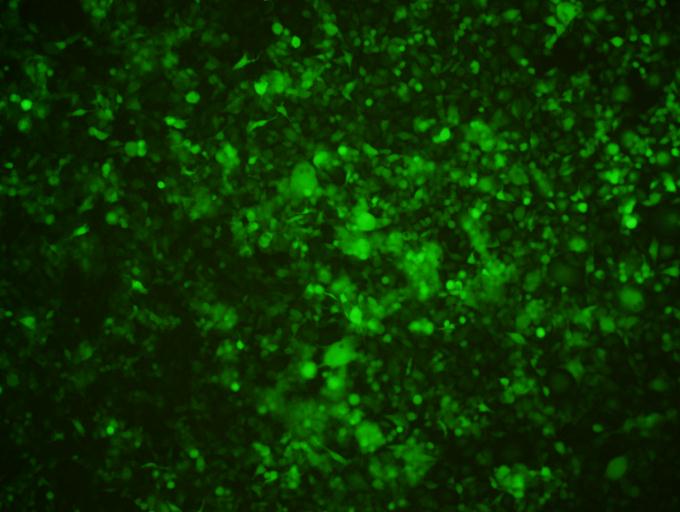

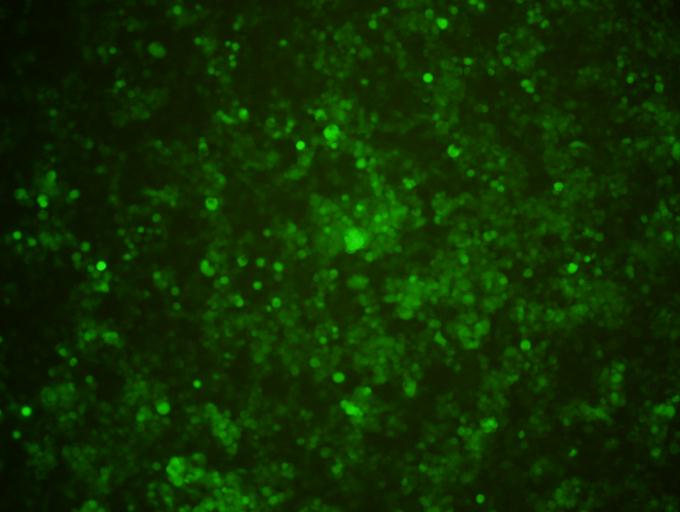

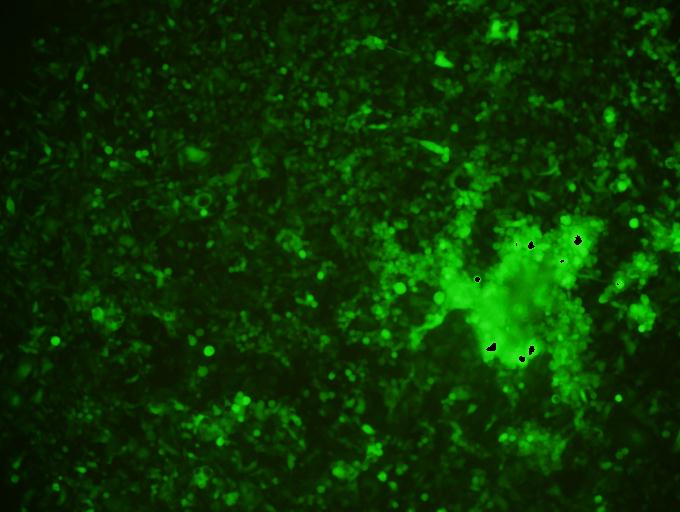

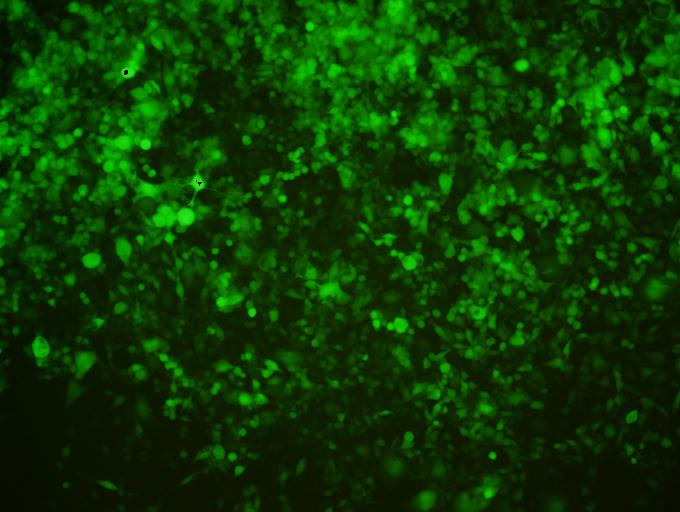


**Mock**

**sh Scb**

**sh Scb**

**sh P2**

**sh Cd**

**sh P3**

**Mock**

**sh P2**

**sh Cd**

**sh P3**

**SGC-7901**

**B**

**
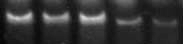

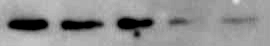
**

**Heparanase**

**Heparanase**

**
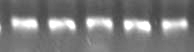
**
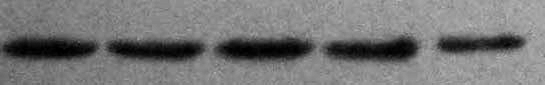


**GAPDH**

**GAPDH**

**PC-3**

**PC-3**

**
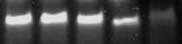
**
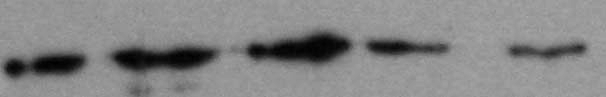


**RT-PCR**

**WB**

**Heparanase**

**Heparanase**

**
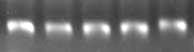
**
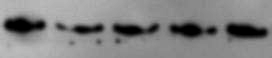


**GAPDH**

**GAPDH**

**EJ**

**EJ**

**
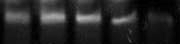
**
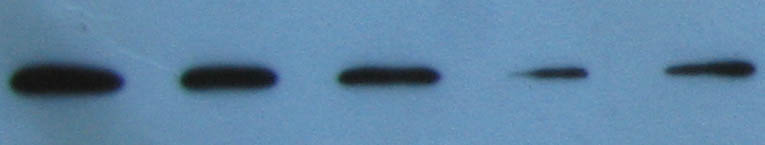


**Heparanase**

**Heparanase**

**
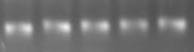
**
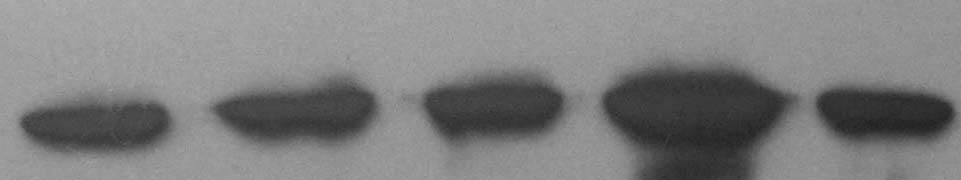


**GAPDH**

**GAPDH**

**SGC-7901**

**SGC-7901**

**C**

**qRT-PCR**

**# #**

**#**

**#**

**Supplementary Figure S4**

**#**

**#**
