# Supplementary material for: Small RNAs Targeting Transcription Start Site Induce Heparanase Silencing through Interference with Transcription Initiation in Human Cancer Cells
Source: PLoS One. 2012 Feb 20;7(2):e31379. doi: 10.1371/journal.pone.0031379 (PMC3282686; doi:10.1371/journal.pone.0031379)
Supplement: Table S1 — Sequences of small interfering RNAs and short hairpin RNAs. (DOC) [file pone.0031379.s005.doc]

**Supplementary Table S1 Sequences of small interfering RNAs and short hairpin RNAs**

| **Small interfering RNAs** | **Sequences** |
| --- | --- |
| siH1 (-174/-155 bp) | 5’-GGGUGAUUUCGUAAGUGAACGtt-3’  3’-ttCCCACUAAAGCAUUCACUUGC-5’ |
| siH2 (-134/-115 bp) | 5’-AGCGAGCAAGGAAGUAGGAtt-3’  3’-ttUCGCUCGUUCCUUCAUCCU-5’ |
| siH3 (-9/+10 bp) | 5’-GGGCGGAGGAAAGGAGAAAtt-3’  3’-ttCCCGCCUCCUUUCCUCUUU-5’ |
| siH4 (+1496/+1515 bp) | 5’-CUCUAAAGAUGGUGGAUGAtt-3’  3’-ttGAGAUUUCUACCACCUACU-5’ |
| siMM | siR-Ribo™ Negative Control |
| siSCb | 5’-GAGAGAGAACGGAGGAGGAtt-3’  3’-ttCUCUCUCUUGCCUCCUCCU-5’ |
| siM31 | 5’- GGGCGCAGGAAACGAGAAAtt-3’  3’-ttCCCGCGUCCUUUGCUCUUU-5’ |
| siM32 | 5’-GGCCGGACGAAAGGAGAAAtt-3’  3’-ttCCGGCCUGCUUUCCUCUUU-5’ |
| DNA3 | 5’-GGGCGGAGGAAAGGAGAAAtt-3’  3’-ttCCCGCCTCCTTTCCTCTTT-5’ |
| siAgo1 | 5’-GAGAAGAGGUGCUCAAGAAuu-3’  3’-uuCUCUUCUCCACGAGUUCUU-5’ |
| siAgo2 | 5’-GCACGGAAGUCCAUCUGAAuu-3’  3’-uuCGUGCCUUCAGGUAGACUU-5’ |
| **Short hairpin RNAs** |  |
| shP2 (-134/-115 bp) | 5’-GATCCAGCGAGCAAGGAAGTAGGACTATGGACATCCTACTTCCTTGCTCGCTTTTTTTGAGCTCA-3’ (sense);  5’-AGCTTGAGCTCAAAAAAAGCGAGCAAGGAAGTAGGATGTCCATAGTCCTACTTCCTTGCTCGCTG-3’(antisese) |
| shP3 (-9/+10 bp) | 5’-GATCCGGGCGGAGGAAAGGAGAAACTATGGACATTTCTCCTTTCCTCCGCCCTTTTTTGAGCTCA-3’ (sense);  5’-AGCTTGAGCTCAAAAAAGGGCGGAGGAAAGGAGAAATGTCCATAGTTTCTCCTTTCCTCCGCCC G-3’(antisense) |
| shCd (+1496/+1515 bp) | 5’-GATCCCTCTAAAGATGGTGGATGACTATGGACATCATCCACCATCTTTAGAG TTTTTTGAGCTCA-3’ (sense);  5’-AGCTTGAGCTCAAAAAACTCTAAAGATGGTGGATGATGTCCATAGTCATCCACCATCTTTAGAG G-3’(antisese) |
| shScb | 5’-GATCCGAGAGAGAACGGAGGAGGACTATGGACATCCTCCTCCGTTCTCTCTCTTTTTTGAGCTCA-3’ (sense);  5’-AGCTTGAGCTCAAAAAAGAGAGAGAACGGAGGAGGATGTCCATAG TCCTCCTCCGTTCTCTCTCG-3’(antisese) |
