# Supplementary material for: Small RNAs Targeting Transcription Start Site Induce Heparanase Silencing through Interference with Transcription Initiation in Human Cancer Cells
Source: PLoS One. 2012 Feb 20;7(2):e31379. doi: 10.1371/journal.pone.0031379 (PMC3282686; doi:10.1371/journal.pone.0031379)
Supplement: Table S2 — Primers sets used for RT-PCR, ChIP and qPCR. (DOC) [file pone.0031379.s006.doc]

**Supplementary Table S2 Primers sets used for RT-PCR, ChIP and qPCR**

| **Primer set** | **Primers** | **Sequence** | **Product size (bp)** | **Application** |
| --- | --- | --- | --- | --- |
| HPA | Forward  Reverse | 5’-GAATGGACGGACTGCTAC-3’  5’-CCAAAGAATACTTGCCTCA-3’ | 261 | RT-PCR, qPCR,  Nuclear run-on |
| GAPDH | Forward  Reverse | 5’-AGAAGGCTGGGGCTCATTTG-3’  5’-AGGGGCCATCCACAGTCTTC-3’ | 258 | RT-PCR, qPCR,  Nuclear run-on |
| HPA/CpG | Forward  Reverse | 5’-GTATTTTTTAAGTGGGTGTGGGT-3’  5’-ATCTTAAACTCACCTAACTACTCCCC-3’ | 296 | BSP |
| HPA/CpG | Forward  Reverse | 5’-AGAGAGTCGGGTAGGCGGGGC-3’  5’-TTTCTCCTTTCCTCCGCCCCGTTACG -3’ | 126 | MSP |
| HPA/CpG | Forward  Reverse | 5’-TAGGAGAGAGTTGGGTAGGTGGGGT-3’  5’-TCTCCTTTCCTCCACCCCATTACA-3’ | 128 | USP |
| HPA  (-6 /+283) | Forward  Reverse | 5’-CGGAGGAAAGGAGAAAAGG-3’  5’-ATGGTGACGGACAGGAACG-3’ | 289 | ChIP |
| HPA  (-183/+13) | Forward  Reverse | 5’-AGTGGGTGTGGGTGATTTC-3’  5’-CCTTTTCTCCTTTCCTCCG-3’ | 196 | ChIP |
| HPA  (-137/+52) | Forward  Reverse | 5’-GAAAGCGAGCAAGGAAGTAGG-3’  5’-TCGAGAGCTCTAGCACTTC-3’ | 189 | ChIP |
| RARβ2 | Forward  Reverse | 5’-GCACGTAGGCTGTTGGTCTTT-3’  5’-GCTGGCTTGTCTGTCATAATTCA-3’ | 61 | ChIP |
| p16 | Forward  Reverse | 5’- TCCTGAAAATCAAGGGTTGAG-3’  5’-GCAAAACTATTCTTTCCTAGTTGTGA-3’ | 102 | ChIP |
| HPA  (exon 1) | Forward  Reverse | 5’- AGTGGGTGTGGGTGATTTC -3’  5’- ATGGTGACGGACAGGAACG-3’ | 232 | Transcript variant |
| HPA  (exon 1) | Forward  Reverse | 5’-CATCTCCGCACCCTTCAA-3’  5’-ATGGTGACGGACAGGAACG-3’ | 249 | Nuclear run-on |
| Ago1 | Forward  Reverse | 5’-GCACTGCCCATTGGCAACGAA-3’  5’-CATTCGCCAGCTCACAATGGCT-3’ | 114 | RT-PCR, qPCR |
| Ago2 | Forward  Reverse | 5’-CGCGTCCGAAGGCTGCTCTA-3’  5’-TGGCTGTGCCTTGTAAAACGCT-3’ | 139 | RT-PCR, qPCR |
| VEGF | Forward  Reverse | 5′-ATGACGAGGGCCTGGAGTGT-3′  5′- CATTTACACGTCTGCGGATCT-3′ | 227 | RT-PCR, qPCR |
| MMP-9 | Forward  Reverse | 5′-GCAGAGGAATACCTGTACCGC-3′  5′-AGGTTTGGAATCTGCCCAGGT-3′ | 196 | RT-PCR, qPCR |
| PCNA | Forward  Reverse | 5’-AAACCAGCTAGACTTTCCTC-3’  5’-TCACGCCCATGGCCAGGTTG-3’ | 273 | qPCR |
| Cyclin D1 | Forward  Reverse | 5’-TGCCGTCCATGCGGAAGAT-3’  5’-CACAGAGGGCAACGAAGGT-3’ | 411 | qPCR |
